# ‘Do you have a future when you are 93?’ Frail older person’s perceptions about the future and end of life – a qualitative interview study in primary care

**DOI:** 10.1080/02813432.2022.2139348

**Published:** 2022-10-29

**Authors:** Anna Olaison, Elisabet Cedersund, Jan Marcusson, Magnus Nord, Annette Sverker

**Affiliations:** a Department of Culture and Society, Linköping University, Norrköping, Sweden; b Department of Culture and Society, Ageing and Social Change, Linköping University, Sweden; c Department of Acute Internal Medicine and Geriatrics, and Department of Health, Medicine and Caring Sciences, Linköping University, Linköping, Sweden; d Primary Health Care Center Valla, and Department of Health, Medicine and Caring Sciences, Linköping University, Linköping, Sweden; e Department of Activity and Health and Department of Health, Medicine and Caring Sciences, Linköping University, Linköping, Sweden

**Keywords:** Frail older persons, future, end of life, primary care, qualitative study

## Abstract

**Objective:**

To explore frail older persons’ perceptions of the future and the end of life.

**Design:**

Qualitative content analysis of individual semi-structured interviews.

**Setting:**

Nine primary health care centres in both small and middle-sized municipalities in Sweden that participated in the intervention project Proactive healthcare for frail elderly persons.

**Subjects/Patients:**

The study includes 20 older persons (eight women and 12 men, aged 76–93 years).

**Main outcome measures:**

Frail older persons’ perceptions of the future and end of life.

**Results:**

The analysis uncovered two main categories: Dealing with the future and Approaching the end of life. Dealing with the future includes two subcategories: Plans and reflections and Distrust and delay. Approaching the end of life includes three subcategories: Practical issues, Worries and realism, and Keeping it away.

**Conclusion:**

This study highlights the diverse ways older people perceive future and the end of life. The results make it possible to further understand the complex phenomenon of frail older persons’ perceptions on the future and the end of life.

**KEY POINTS**
The study found that older persons described their future as contradictory- with a broad spectrum of approaches, where some wanted to deal with these subjects and others wanted to ignore them.
•Older persons that consciously planned for the future had tactics that often were related to goals that functioned as motivators to live longer.
•Those who adopted a more passive approach did not think about what the future might hold in terms of losing autonomy and deteriorating health.
•Older persons that approached end of life in a more proactive way wanted to plan practical arrangements around death but often found it hard to address this issue with relatives.
•Those older persons that had a more passive approach to end of life preferred not to think about those issues, and some explicitly stated that they did not want to address the final period of life.

## Introduction

The past number of years has witnessed a shift in health care in many countries, from acute and institutional care to home health care and home care services for older persons in need of care and support [1,2]. These changes have resulted in a large number of frail older persons living at home and being dependent on health care provided by primary care and community-based home care [3,4]. In Sweden, primary care coordinates actors in the welfare system involved in elder care. However, a changing elder care requires more focus on developing primary care with a holistic view on the health of older people [5,6]. According to the National Board of Health and Welfare in 2021 in Sweden, approximately 236,000 older persons aged 65 and older received home health care. Out of these, 57% of women and 52% of men had both home health care and home care services [7]. Also, frail older persons living at home experience their health as poor to a much greater extent if they live at home compared to if they live in special housing [8]. Among those receiving home health care, 24% rate their health as good while the corresponding proportion among those in special housing is 40% [8]. An older person living at home with complex health care needs usually has reduced ability to self-coordinate their care and services due to declining health [9]. Frail older persons also run a greater risk of having fewer opportunities to discuss their relationships and thoughts about future care and end of life [9,10].

Knowledge and understanding of frail older persons’ perceptions about the future and end of life are consequently fundamental for staff work in primary care to enable personalised and appropriate care. Although, previous research has found that older persons have thoughts about the future and in many cases want to discuss different aspects of end of life [9,10] this has not been addressed to a wide extent in health and primary care research. Several studies have shown that older persons often do not have the opportunity to discuss questions related to the future and end of life [11,12]. The present study contributes to this area by exploring this theme using semi-structured interviews in a group of frail and pre-frail elderly persons. That is, the study aimed to explore frail older persons’ perceptions on the future and the end of life.

Before reporting the empirical results about the older persons’ perceptions of the future and end of life, we will provide a short overview of recent research about older persons’ experiences of frailty in later life and their thoughts about the future as this is our central theme.

### Research on frail older persons and how they may perceive the future

Frailty in old age is experienced as a variety of losses including physical, psychological, social, and existential [13]. Living at home is strongly connected to independence and autonomy for older persons [14,15] as the home is where they develop and sustain contacts with their physical environment, habits, and social networks [16]. In addition, older people want to maintain independence in old age without being a burden on others [17,18]. Being frail and living in the tension between autonomy and dependency is a complex emotional situation as older persons must attempt to acknowledge the consequences of life, specifically the inevitability of loss and death [19]. However, homebound frail older people see themselves as living with little identification of or support for their thoughts about the future [16]. This lack of encouragement to express thoughts and reflections about living and dying could be linked to the experience of frailty that contests the dominant cultural and welfare practices and policy frameworks that only view frail older persons as dependent on care [15,20]. This focus on dependence and decline discourages thoughts about the future. Improving the experiences of frail older people living at home must involve addressing the problematized nature of old age and ageing while acknowledging cultural aspects of living and dying. That is, research needs to consider the challenges of the increased number of frail older persons living at home and their experiences as they enter their end of life [19,21]. Here, we bridge this knowledge gap by focusing on frail older persons’ perceptions about their future and its consequences for their experiences with end of life.

### The intervention project proactive healthcare for frail elderly persons

The initiative Proactive healthcare for frail elderly persons was implemented as part of an intervention study in the Region Östergötland [6]. In this intervention study, the effectiveness of a primary care intervention consisting of comprehensive geriatric assessment and individually tailored follow-up over two years was compared with usual primary care [6,21]. Participants were selected with a prediction model that uses electronic medical records to identify older persons over 75 years of age with a high risk for hospitalisation. The project included several sub-studies of the care model, which used the cumulative deficit model of frailty, expressed in the Clinical Frailty Scale developed by Rockwood and Mitinsky [6,22]. This is also the frailty model we have used in this sub-study. The sub-study reported in the present article concerns older persons’ views on participation in care and social networks with a focus on their perceptions of their future and end of life.

## Materials and methods

### Design

This study had a qualitative design using qualitative content analysis. All participants provided informed consent.

### Participants

The reported sub-study included 20 older persons who were living in three small or middle-sized municipalities in the Region of Östergötland in Sweden. The participants consisted of eight women and 12 men, aged 76–93 years. These participants represented all nine primary health care centres that were active in the intervention study.

A purposive sampling strategy was used [23]. All participants were recruited from the lists of participants that were selected with the prediction model for the intervention. A nurse from each of the primary care centres provided the research team the names of four participants (two men and two women). A time selection principle was also used to choose patients: the first patients treated at the primary care centres during November 2017 were selected.

The potential participants were sent a letter with information about the study, and they were after that also contacted by telephone by one of the researchers, and asked about participation. A time and place for the interview was arranged. In all, 20 participants agreed to take part in an interview. Of the 20 participating, 10 of them consented to the researchers having access to their patient records. In the patient records there were 589 registered diagnoses of the 10 persons in a five-year period 2013–2017, which gives a mean of 59 diagnoses per person during a five-year period. The diagnoses comprised everything from those of a more psychological nature such as anxiety, to more serious medical diagnoses such as cancer. This gives an indication of the variety and complexity of the different health problems of the older persons. In addition to that, we could only get access to the information that the participants themselves choose to disclose about their diagnoses under the interviews

### Data collection

The interviews were semi-structured and used open-ended questions. The interview guide can be sent on request. Three of the researchers with experience in qualitative research from different scientific disciplines, such as ageing, and social work research, conducted the interviews. After an opening presentation of the project, the participants were asked about their experiences with ‘everyday life/care’, ‘autonomy/own impact on health care/services’, and ‘the future and end of life’. This study presents the participants’ perceptions about the future and end of life. Overall, the interviewers encouraged the participants to freely voice their thoughts and experiences. At the end of the interview, the participants were asked if they wanted to add anything extra. The interviews lasted between 27 min and 114 min and were digitally recorded with the participant’s permission and were transcribed verbatim by a skilled external secretarial service. The data were collected between December 2017 and July 2018.

### Analysis

The material was analysed using conventional content analysis [24]. This method was chosen, as it is appropriate in analyses aiming to describe a phenomenon [25], in our case perceptions of the future and end of life by older persons. Content analysis is also useful when researchers want to avoid preconceived categories and instead allow the categories to emerge from the data [24,25]. Initially, four researchers separately read all 20 transcribed interviews to obtain a general overview of the information regarding participants’ views of the future and end of life. The transcribed text containing information about the future and the end of life was abstracted, condensed, and sorted into groups, which included the original quotations. After the initial reading and the first analysis, the last author more systematically worked through the material, focusing on the participants’ experiences of the future and the end of life. Validity was built into the analysis by frequently testing the body of results against new data until saturation was reached i.e. the evolving pattern was not changed by new data. Subsequently, the research group discussed categories and subcategories until consensus was reached. Table 1 lists examples of the meaning units, condensed meaning units, codes, subcategories, and categories.

**Table 1.** Examples of the process of analysis with meaning units, condensed meaning units, codes, subcategories, and categories.

Meaning unit	Condensed meaning unit	Code	Subcategory	Category
‘…of course, when you’re 80 you do that… Then I think like…well, what is it going to be like in the future. And I want to… Well, want to, that is, I hope that we will be able to keep living here for a few more years. And that we can live together…No, not ten. I don’t think so but let’s say five. Hopefully’.	Thinks about the future. Wants to keep living with his wife in their flat for a few more years. Hopes for five years. Thinks that it is obvious that you think about the future when you are 80 years old. Hopes to stay in the apartment for a few more years and live together with his wife.	Long future	Plans and reflections	Dealing with the future
‘…So I have…it sounds a little bizarre…I think that, no but I’m going to die suddenly. It…so I have…what do you call it…orderly as I am, I have written it down and I have a clear idea about how it should be conducted and how it should be taken care of. And I’ve written that in documents that my daughter will have access to’.	Has written down how it is to be done and taken care of. Has written documents about his funeral.	Funeral	Practical issues	Approaching the end of life
‘I don’t think that much about the future. I know of course what it entails, the future. It’s only one thing. Nothing else, is there?’	Does not want to talk about the future. There is nothing to talk about. She knows what the future holds.	Death awaits	Worries and realism	Approaching the end of life

## Results

This study identified two categories: *Dealing with the future* and *Approaching the end of life. Dealing with the future* comprised of the two subcategories *Plans and reflections* and *Distrust and delay. Approaching the end of life* included the three subcategories *Practical issues*, *Worries and realism* and *Keeping it away* (Table 2).

**Table 2.** Perceptions about the future; categories, subcategories, and codes.

Category	Subcategory	Code
Dealing with the future	Plans and reflections	Changed way of thinking
		Unchanged way of thinking
		Long future
		Short future
		Expectations of longevity
		Worries
	Distrust and delay	Procrastinates thoughts about the future
		Expresses no thoughts about the future
Approaching the end of life	Practical issues	The will
		The funeral
		Who wants to listen
	Worries and realism	Death awaits
		A dignified end
		A sudden death
	Keeping it away	Does not think about the final period of life
		Does not want to think about the final period of life
		Changes the subject

### Dealing with the future

#### Plans and reflections

Several participants explained that they had changed their way of thinking about the future. They described their thoughts about the future – i.e. what their last years of life will be like. One participant stated that thoughts about the future were different now compared to ten years ago. Thoughts about the future were also dependent on what happens in life, for example; a husband’s illness brought up thoughts about the future. The participants also expressed thoughts about the future more often as their own illnesses progressed. One participant described her situation as follows:

It’s now that I have become more ill than I have been. […] I used to be able to go outside. Now I don’t even go outside the door […] that makes me think in a different way about the future, I know what the future means, that there is only one thing that matters. […] something else does not apply. (Interviewee no. 18)

Other participants did not think about the future changing – i.e. they had an unchanged way of thinking. They took a more passive approach to the future. The diseases had not changed the participants’ thoughts about the future.

One participant claimed that he did not think differently about the future:

Since I get by so well … yeah … I tend to procrastinate these things […] I just say, acknowledge that I know that it will come … it crops up at times you know. To that extent I’m prepared. But it’s nothing that I go and think about, I would say. (Interviewee no. 12)

A long future included statements with a perspective of between one week and up to five years. The participants talked about plans for the future, often with the goal of fulfilling their plans. When one participant thought about the future, he hoped that there is a future five years ahead:

[…] of course, when you’re 80 you do that. […] Then I think like … well, what is it going to be like in the future. And I want to … well, want to, that is, I hope that we will be able to keep living here for a few more years. And that we can live together […] let’s say five [years], hopefully. (Interviewee no. 11)

For participants with a longer-term perspective about the future, thoughts about children and grandchildren were strong motivations to live. One participant described this desire:

I got cancer […] now it’s over … and then I thought like this … no, it shall not be over. No. I’ll figure this out […]so come here, my grandchild […] he’ll be 13 now […]and they caressed my hands … grandma, when I graduate you probably won’t be alive. No, I probably won’t, I said, just like that. Then I came home and thought like this … how old will I be when he graduates? Well, I calculated and … I’m 93. I’ll make it, I thought. (Interviewee no. 2)

The participants also stated that they made plans for trips with their relatives. Travel seemed to be important to some of the participants when they thought about the future in a longer-term perspective. Some participants had an even shorter future perspective. They described that you take one day at a time and that they feel a thankfulness for each day. The participants stated that the future perspective could change quickly if you are ill.

Other participants reflected on and had expectations of longevity. They wanted to live until they were100 years old. Several stated that they had relatives who have grown old, and then they had their own expectations on longevity.

The participants stated different worries related to the future; that they worried about their finances and how they would get by when their partner died. Worries could also be expressed in terms of uncertainty about the future: one hopes for a future, but it feels uncertain. At the same time, many expressed that they are not worried about or scared of the future but accept whatever happens. One participant stated that she were not scared of the future; she takes one day at a time and as long as she could get out of bed in the morning and take care of herself, she were not concerned about the future. At the same time, she expressed a concern that is linked to deteriorating health:

What I worry about the most, I suppose it is if you get another stroke, right, and then you sit in a wheelchair and can’t talk or anything and can’t move. That would be horrible. Then it’s better to die. Absolutely. I think so. Other than that, I’m not afraid of the future. (Interviewee no. 13)

#### Distrust and Delay

Many participants actively avoided thinking about and discussing the future. The code procrastinates thoughts about the future implied that one does not think about the future. These participants did not make plans for the future and did not want to talk about it. The participants described an uncertainty and out of fear of disappointment they did not want to think about the future. Some expressed that they did not have a future in old age. One participant claimed that she tries to make the most of each day:

I do my best. This is going to be a good day, almost, and not that much further ahead. That’s how we do it. And not plan and not think. Because then you’ll be disappointed. (Interviewee no. 14)

The code does not express any thoughts about the future described sentiments where the participants took on a more passive approach towards the future and let time just go by and did not want to talk about it either. Participants stated that they did not want to think about losing autonomy until it is necessary. A feeling was expressed about being unsuccessful if you had to receive care. Simultaneously, the participants were also aware that if you had care, you are often positive about receiving it. However, most of the participants wanted to manage independently for as long as possible, otherwise they felt unsuccessful.

These participants did not think about the future and stated, ‘one does not have a future in old age’ so one can just ‘keep going until the bitter end’. One participant expressed that he and his wife have had a lot of company and a large circle of acquaintances, but they were almost the only ones left and had a passive approach to life:

We have a daughter and then we have the deceased daughter’s two children. And then they in turn have two little children that we can’t really cope with. They are three and four years old. We don’t see them that much. But we like them and all, but … now we sit here and let life go by. (Interviewee no. 3)

### Approaching the end of life

#### Practical issues

When participants talked and described their thoughts about planning for the last period in life, some did so in a more active way. Some statements were related to the fact that the participant took a more active approach to planning for the last days of life, while others were of a purely practical nature. The participants stated that it was important to write a will, to clarify to relatives their wishes about their home, money and household goods. Another more active approach to the last days of life were that you think about your funeral and clarify how you want the funeral to be. The participants stated that they had written down their wishes in various documents, and that relatives knew about these and where they were stored.

In the following quotation, the participant described what he and his wife had discussed:

[…] and she has written down what we want it to be like. The funeral not at the church with a whole bunch of people, but … well the chapel down here and verses … it’s in the third drawer in my desk. And the children know about this. (Interviewee no. 1)

The code who wants to listen described how participants wanted to plan for the final period of life but they found it difficult to talk about or to address this issue with their relatives.

There were thoughts and reflections about not wanting to become dependent on care during the last period of life. The participants said that they wanted to stay in their apartment, that they did not want care in special housing. The participants thought that you should talk to your children and their relatives about this, and one participant described this situation in the following way

I have tried to talk to my children, but they don’t want to listen […] yes but it’s so strange. It’s the one thing that you know will happen. But you never talk about it […] but as I said, when none of the kids want to listen, what do you do? (Interviewee no. 20)

The participant also stated how she had tried to talk to her children about the final period of life, about her wish to keep living in her home, but described that she got no response from her children.

The participant said that she had a desire to have a conversation with the children about the last period of life, about where she wanted live, and the desire to stay in her own home. However, the children did not want to address these issues, and they left it to her to handle on her own.

[…] I have told them that they will have to carry me out. They haven’t said anything about it. They just dumped one of these Fonus [funeral home] papers where I can write down how I want it. (Interviewee no. 18)

#### Worries and realism

When the participants reflected on death and the end of life, many described an acceptance and acknowledgement of the fact that life is ending. It was not perceived as something to brood over or discuss. In the code death awaits, thoughts about knowing what will happen and what the future entails were described. The participants expressed thoughts about life after this, and that they thought a lot about death. The thoughts about death came more often as they get older and more ill. You know how things are looking and what the future holds. This participant knows where things are going:

I know of course what it entails, the future. It’s only one thing. Nothing else, is there? So that’s why it must be so. (Interviewee no. 18)

The participant reflected on death from a different perspective. He did not brood deeply over the future but rather described that he just keeps going.

That’s why I think like this, I’ll keep going until the bitter end. […] I am a deep thinker in this regard. I don’t brood for a minute. I don’t’. (Interviewee no. 4)

Thoughts on what the final period life should be like were largely about a dignified end. Many were worried that they would not be able to die at home and not receive good care in case they needed to move to a care home.

Losing one’s autonomy or enduring pain were frightening. Many expressed hopes of a sudden death. One participant reflected on what it means to die suddenly and that the final period of life should go fast:

[…] I wouldn’t want to end up lying in a bed for five to ten years. That’s horrible, isn’t it. It’s best if you can die suddenly. That’s just how it is […] I think it would be nice if I could get to be here and pass away in my sleep in my own bed. (Interviewee no. 13)

#### Keeping it away

In several of the interviews, the participants expressed that they did not think about the final period, they did not want to think about the final period, and in some cases, they entirely avoided answering questions on the final period of life, for example, by changing the subject.

Not thinking about the final period of life could mean that one relinquished the responsibility to a close relative.

That some of the participants did not want to think about the last period of life is reflected in the dialogue below with the interviewer. This participant described that she did not want to think about the final period of life. She just wanted it to go fast. She did not think that her children knew that this is how she feels:

Interviewer: The final period of life, have you thought about that?

Interviewee: No.

Interviewer: No. And you don’t want to?

Interviewee: Just that it goes fast…one can wish.

Interviewer: Do your children know that this is what you want, too?

Interviewee: No, I don’t think so.

Interviewer: No. You never talk about it?

Interviewee: No, we don’t.

(Interviewee no. 15)

When the interviewer asked whether she thought about the end of life, the participant stated the following: ‘Not in everyday life. It’s all action. There’s the Nobel on Sunday. We always watch the Nobel party. Here, she changed the subject when the interviewer asked about thoughts on death and the end of life. The participant clearly did not want to talk about the future or the final period of life.

## Discussion

### Main findings

This study has described the older person’s perceptions about the future and end of life. The participants described their future as contradictory- as something they viewed in different ways, with a broad spectrum of approaches and different horizon expectations. The future could mean a shorter perspective or expectations or longer perspectives with clear expectations for life expectancy with a desire for a long life and plans for future events. Those participants that more consciously planned for the future had strategies that were often related to goals that functioned as motivators to live longer such as family events or scheduled trips. However, some participants did not express any thoughts about the future or wanted to postpone or delay thinking about the future. This more passive approach consisted of not thinking about what the future might hold in terms of losing autonomy and deteriorating health, and instead being in the present and enjoying taking one day at a time.

The topic of end of life was approached by the participants in either a more active or passive way. Those older persons that approached end of life in a more proactive way wanted to arrange practical things such as the will or preparing the funeral. Recurring findings in the study were that some participants really wanted to talk about and plan the end of life period, as well as engage in planning around death, something that the relatives sometimes did not want to discuss.

When reflecting about death, some of the participants had an accepting approach, hoping for a dignified end where they preferred to live independently at home for as long as possible. Others expressed hope for a short final period of life with a sudden death. Those older persons that had a more passive approach to end of life preferred not to think about those issues and some also explicitly stated that they did not want to talk about the final period of life.

### Comparison with existing literature

This result is in line with studies [9] where the future is either seen with confidence, with the hope of living long and experiencing as much as possible, or seen with trepidation, with the belief that the rest of life is pointless or a waste of time. Furthermore, several studies also describe how older people may wish for a long life and look forward to future activities in everyday life [14,20], results also noted in this study.

Thoughts about the future were also connected to independence and autonomy associated with living at home. These results are in line with studies highlighting the importance of balancing independence and an active life with being frail and dependent on care [17,18]. This study also described the frightening feeling of losing autonomy and becoming completely dependent on care, results that are in line with previous studies [14,15]. Living a life with integrity meant continuing to live at home with a feeling of meaningfulness. Talking about the future also involves how approaching the end of life and the participants related to this in different ways. Active planning was an opportunity to guide and inform their relatives about their preferences before it was too late. These participants wanted to talk to their relatives about their funeral plans, including what they did not want their relatives to do. Many participants found that their relatives did not want to address or talk about death as they found that their relatives often changed the subject or ignored the topic of end of life. These findings agree with the results of a previous dissertation [10]. In addition, these results are in line with other studies’ conclusions that older persons tend to plan their own death in terms of, for example, funeral arrangements. However, planning for one’s own death is more unusual [12,26] even though dying is what older persons often are most worried about [15]. Therefore, it is increasingly important to give older people the opportunity to talk and think about death. If this opportunity is not given, there is a risk that older persons will be left alone to think about and address their worries and anxieties about death [27]. It is also important to have conversations about the family dynamics of approaching the end of life to ensure that older persons’ preferences are respected [11].

### Meaning of the study

The results from this study make it possible to further understand the complex phenomenon of frail older persons’ perceptions of the future and the end of life. The findings reiterate themes identified in prior research: relating to and reflecting on the future and the end of life can be emotive and stressful topics [11,20] that often can be connected to declining health and a frailty identity [9,12] irrespective of whether the older persons will discuss the issues. The findings from this study suggest that the future and end of life are topics that older persons want to discuss; that is, they want to address the if, and how, questions. Giving older persons the opportunity to address these issues in a way that they prefer can be meaningful and improve quality of life. Therefore, healthcare staff must continuously and senistively address older persons’ thoughts about the future.

### Clinical implications

The results highlight the diversity of ways older persons relate to the future and end of life. In light of this, it is important to address an individual’s own worries and expectations to ensure a patient-centred approach. Therefore, the continuity of care is very important as this would make it easier for older persons to discuss these important matters, which often requires several discussions.

Talking about the future can be a challenging issue for older persons. Primary care teams must have a permissive approach and be prepared that the individual and his or her relatives want to raise and discuss issues concerning the future and the last days of life. Some participants in this study were not eager or able to discuss the future, issues that call for sensitivity among healthcare staff. At the same time, staff need to give their patients an opportunity to talk about the future and end of life. Proactive planning of care requires a discussion between the patient and the care staff about the patient’s future care to meet the needs of frail older persons. Communication about older persons’ future requirements needs to be adapted to the older persons’ thoughts and beliefs. That is, these discussions need to be tailored to each person’s needs and capabilities. As a result of this, interventions for frail older persons should include ways to more proactively involve staff in primary care in communication training so they can more effectively address views and questions on the future and end of life in ways tailored to an individual’s specific needs.

### Strengths and limitations

This study included interviews with 20 older persons living at home. The interviewees varied in age, gender and marital status. Many contacted persons declined to be interviewed (*n* = 17); this limitation might mean that those persons who participated could have been more motivated to talk about their situation. That is, the sample might not reflect thoughts from the frailest older persons. Another limitation includes a potential for sampling bias by using a purposive sample as an inclusion criterion. As our sample was selected from a patient list at the primary health care centres this could inevitably include a level of self-selection of patients by the nurses, resulting in a possibility that patients recruited were considered as suitable candidates to interview. The data collection was conducted through semi-structured interviews, which contributed to spontaneous accounts from the older persons on what they decided to share about the topics. This can be considered a strength in terms of the credibility of the study. Another strength was that the authors, as independent researchers with no connection or decision-making power over older persons care and services, conducted the interviews. This arrangement might have contributed to them being able to express their thoughts and concerns more freely. In addition, the participation of four of the authors in the analysis process reduced the risk of misinterpretation of the data. The research group had continuous discussions during the analysis process, which strengthened the reliability of the results.
